# Slow motion

**DOI:** 10.7554/eLife.24792

**Published:** 2017-02-14

**Authors:** Naoshige Uchida, Jeremiah Y Cohen

**Affiliations:** 1 Center for Brain Science, Department of Molecular and Cellular Biology, Harvard University, Cambridge, United Statesuchida@mcb.harvard.edu; 2 Solomon H Snyder Department of Neuroscience and the Brain Science Institute, The Johns Hopkins University School of Medicine, Baltimore, United Statesjeremiah.cohen@jhmi.edu

**Keywords:** serotonin, dorsal raphe nucleus, locomotion, optogenetics, Mouse

## Abstract

Optogenetic stimulation of serotonin neurons in the dorsal raphe causes mice to move more slowly without causing any apparent motor deficits or anxiety-like effects.

**Related research article** Correia PA, Lottem E, Banerjee D, Machado AS, Carey MR, Mainen ZF. 2017. Transient inhibition and long-term facilitation of locomotion by phasic optogenetic activation of serotonin neurons. *eLife* **6**:e20975. doi: 10.7554/eLife.20975

Of all the neurotransmitters known to have a role in the nervous system, serotonin is one of the most mysterious. It is thought to be involved in a number of brain functions (including mood, eating and sleep), and it has also been implicated in depression and other psychiatric disorders. However, compared to other neuromodulators such as dopamine (Schultz et al., 1997), there is much that we don't know about serotonin.

One might think that the development of optogenetic techniques that can stimulate specific neurons would have clarified the situation, but a clear picture has yet to emerge. For example, one study found that the stimulation of serotonin neurons in a region of the brainstem called the dorsal raphe was rewarding, meaning that it encouraged the mice to repeat certain forms of behavior (Liu et al., 2014), but later studies found that such stimulation was not rewarding, and that these neurons were instead linked to functions such as patience (the ability to wait for upcoming reward) or response inhibition (the ability to reduce speed of movement; Fonseca et al., 2015; McDevitt et al., 2014; Miyazaki et al., 2014). Another study showed that activation of serotonin neurons projecting to a specific part of the brain (the BNST) caused anxiety-like behaviors (Marcinkiewcz et al., 2016).

Now, in eLife, Zachary Mainen of the Champalimaud Centre for the Unknown and co-workers – including Patrícia Correia and Eran Lottem as joint first authors – report the results of a series of very careful experiments on mice that help to clarify the roles of serotonin (Correia et al., 2017).

First, in experiments in which the mice were confined in an arena, the researchers showed that the mice spent more time in the area of the arena where a laser was used to stimulate the dorsal raphe serotonin neurons. However, the mice did not enter the stimulation area more often; rather, they spent more time in this area because they slowed down when their serotonin neurons were stimulated by the laser. Indeed, regardless of where the animal was in the arena, or how fast it was moving, activation of these neurons immediately slowed their spontaneous locomotor activity, causing a 90% drop in speed within 1 second. The same stimulation did not interfere with other behaviors (such as rearing, digging and grooming), but it did lead to an increase in resting behavior. Correia et al. further showed that the stimulation of these neurons did not impair the performance of the mice in a skilled motor task (which involved the mice learning not to fall from a rotating rod). Furthermore, the pattern of steps during motion was not altered. These results show that stimulation of the dorsal raphe serotonin neurons reduces speed without affecting motor behaviors generally.

In an effort to determine why stimulation causes the mice to reduce speed, Correia et al. employed a well known assay of anxiety that exploits the fact that mice usually avoid the center of an open space, and spend more time at the periphery. This preference can be reduced by anxiolytic drugs and is thought to originate from the innate anxiety of the mice in an open environment. Correia et al. found that the reduction in speed was not due to increased anxiety. However, they also demonstrated that the inhibition of locomotion was greatly reduced when the animal had already started to move in the pursuit of a reward, suggesting that the effect can be overcome by a strong motivation to move. Finally, Correia et al. showed that repeated stimulation of dorsal raphe serotonin neurons over a period of days gradually increased locomotor activity (in contrast to the decrease that occurred on shorter timescales).

The exact relation between this latest work – which, in short, shows that serotonin is involved in response inhibition – and previous studies remains to be clarified. The rewarding effect observed by Liu et al. (that is, the observation that optogenetic stimulation caused the mice to visit the stimulation area more often and also spend more time there) may be due to the fact that they also stimulated other types of neurons (see also McDevitt et al., 2014).

Likewise, the differences between the present study and the work of Marcinkiewcz et al. – who found that the activation of serotonin neurons caused anxiety-like effects without affecting locomotor activity – may be because the latter stimulated a specific population of serotonin neurons projecting to the BNST, whereas the former stimulated a larger population. Nonetheless, this issue warrants further investigation because Marcinkiewcz et al. also observed some reduction in locomotion. Can the patience effect be explained by this reduction? The answer is not so simple. Correia et al. showed that when the animal had already initiated a reward-seeking behavior, locomotor inhibition did not occur as easily. Locomotor inhibition might not, therefore, manifest in a decision-making paradigm where the animal is strongly motivated to perform. Despite its common usage in everyday life, patience is likely to be controlled or affected by multiple psychological processes, and further work is needed to understand the relationship between the functions of serotonin in patience and locomotor inhibition.

Finally, the interpretation of optogenetic stimulation experiments requires some caution. Techniques such as optogenetic tagging (Cohen et al., 2015; Li et al., 2016; Liu et al., 2014) and fiber fluorometry (Matias et al., 2016; Li et al., 2016) have begun to provide data on the activity of specific serotonin neurons. These experiments generally find diverse firing patterns in serotonin neurons. It is therefore not clear if optogenetic stimulation, which activates neurons uniformly, leads to patterns of activity that are similar to those observed in natural conditions.

Even more importantly, we need to know when and how neurons change their activity, and if different populations of neurons change in the same or different ways. A previous optogenetic experiment suggested that serotonin neurons located in the median raphe (which is underneath the dorsal raphe) are related to anxiety, while serotonin neurons in the dorsal raphe are related to locomotor activity (Ohmura et al., 2014). This highlights the need to take the potential diversity of serotonin neurons into account when designing experiments.

Serotonin remains a mysterious molecule, but in setting a new standard for interpreting behavioral results, Correia et al. have taken an important step toward understanding its functions.
